# Reverse cardiac remodeling in patients undergoing combination therapy of transcatheter mitral valve repair

**DOI:** 10.3389/fcvm.2023.1029103

**Published:** 2023-02-15

**Authors:** Hiroaki Yokoyama, Tobias Friedrich Ruf, Martin Geyer, Alexander R. Tamm, Jaqueline Grace Da Rocha E Silva, Theresa Ann Maria Gößler, Julia Zirbs, Ben Schwidtal, Thomas Münzel, Ralph Stephan von Bardeleben

**Affiliations:** ^1^Department of Cardiology and Catheterisation Laboratories, Shonan Kamakura General Hospital, Kamakura, Japan; ^2^Department of Cardiology, Cardiology I, University Medical Center Mainz, Johannes Gutenberg University Mainz, Mainz, Germany

**Keywords:** mitral regurgitation, mitral annuloplasty, COMBO therapy, reverse cardiac remodeling in patients with dilated cardiomyopathy, TMVr, transcatheter

## Abstract

**Aims:**

For patients with severe mitral valve regurgitation (MR), different kinds of transcatheter mitral valve repair (TMVr) exist, targeting the leaflets, annulus, and chordae. The concomitant combination (COMBO) therapy of TMVrs is rarely used as treatment, and there are very few publications about this therapeutic strategy. We evaluated the effect of COMBO-TMVr on the cardiac left chambers and clinical data, including survival.

**Methods:**

We included 35 patients at high risk who underwent concomitant sequential transcatheter mitral valve edge-to-edge repair (M-TEER) and another TMVr for severe MR in our hospital between March 2015 and April 2018. Of these, 13 had adequate follow-up transthoracic echocardiography (TTE) up to around 1 year after the procedure.

**Results:**

Survival for all patients was 83% at 1 year, 71% at 2 years, and 63% at 3 years, respectively. In the 13 patients with adequate TTE follow-up, M-TEER plus either Cardioband (*n* = 4), Carillon Mitral Contour System (*n* = 7), or Neochord (*n* = 2) were used, respectively. Ten patients had secondary, and three patients primary MR. After 1 year, changes [median (Q1, Q3)] of left ventricular (LV) end-systolic diameter of −9.9 cm (−11.1, 0.4), LV end-diastolic diameter of −3.3 cm (−8.5, 0.0), LV end-systolic volume (LVESV) of −17.4 mL (−32.6, −0.4), LV end-diastolic volume (LVEDV) of −13.5 mL (−15.9, −3.2), LV mass of −19.5 g (−24.2, −7.6), and left atrial volume (LAV) index (LAVi) of −16.4 mL (−23.3, −11.3) were observed. A significant reduction was also seen in the change ratios of LVESV, LVEDV, LV mass, and LAVi, respectively.

**Conclusion:**

We found that COMBO therapy of TMVr seems feasible and may support reverse remodeling of left cardiac chambers during 1 year after the procedure in a cohort of patients at high risk.

## Introduction

Mitral regurgitation (MR) is among the most common valvular heart disorders (1), and MR, even isolated, is associated with heart failure, and excess mortality (2). For patients with symptomatic severe MR, who are at prohibitive risk for cardiac surgery, current guidelines recommend mitral transcatheter edge-to-edge repair (M-TEER) as class IIa therapy in secondary MR (SMR) and class IIb in primary MR (PMR) (3), providing reductions in mortality and heart failure hospitalizations (4–6). Also, other types of transcatheter mitral valve repair (TMVr) targeting the mitral annulus, the mitral valve chordae, as well as the mitral valve leaflets have become feasible and safe alternatives in high risk patients suffering from severe MR (7).

As severe MR is the result of the complex interplay between the single components of the mitral valve apparatus, i.e., chords, leaflets, and annulus, respectively, it comes as a surprise that the TMVrs addressing these single components are rarely combined in one procedure (“COMBO-TMVr”) in order to achieve a more integrated approach (8–11).

In this study, we evaluated the effect of different approaches using COMBO-TMVr for the treatment of severe MR on the cardiac chambers. In detail, M-TEER using MitraClip (Abbott Laboratories, Abbott Vascular, Santa Clara, CA, USA) was either combined with Carillon Mitral Contour System (CMCS; Cardiac Dimensions, Kirkland, WA, USA) (12, 13), or Cardioband (Edwards Lifesciences, Irvine, CA, USA) (14), respectively, as annuloplasty, designed to reduce the mitral annulus to correct SMR, or with the transapical Neochord (NeoChord Inc., St. Louis Park, MN, USA), designed to deliver artificial chordae tendineae for the repair of PMR (15).

## Materials and methods

### Study population

We retrospectively evaluated the patients who were treated sequentially for symptomatic significant MR using COMBO-TMVr with M-TEER and another device. The timeframe for data collection was set from March 2015 to April 2018. Data on Mortality was acquired for all patients. Patients were also included for echocardiographic analysis, if adequate echocardiographic follow-up data were available at around 1 month and around 1 year. The heart team evaluated all patients to be stable, eligible for transcatheter therapy, but ineligible for cardiac surgery. While individual factors were considered, this evaluation was strongly based on the logistic EuroScore, and values above 6% were considered “high risk” for cardiac surgery (16, 17). The study was approved by the local ethics committee (2019-14692).

### Procedures of transcatheter mitral valve repair

**FIGURE 1:**
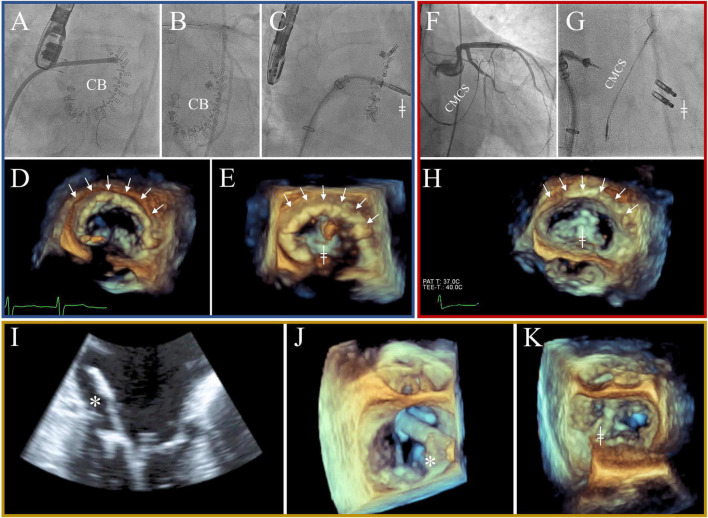
All three combination (COMBO) strategies with blue: Cardioband + mitral transcatheter edge-to-edge repair (M-TEER) **(A–E)**, red: Carillon Mitral Contour System (CMCS) + M-TEER **(F–H)**, and orange: Neochord + M-TEER **(I–K)**. Blue: Cardioband is implanted using fluoroscopy [A + B; “Cardioband (CB)” and arrows] and 3D echocardiography **(D)**. Intermittently, unimpaired flow of left circumflex coronary artery (LCX) is checked **(B)**. Red: CMCS is implanted using fluoroscopy (F + G). Intermittently, unimpaired flow of LCX is checked **(F)**. Note the pull of the CMCS in the left atrium displayed in 3D echocardiography (arrows). Orange: Neochord is implanted using echocardiography [2D: **(I)** and 3D: **(J)**]. Please note the open jaws of the NeoChord Implanting system, ready to grasp the flailing posterrior mitral leaflet (*). MC is implanted second to either Cardioband **(C,E)**, CMCS **(G,H)**, or Neochord **(K)**, using both fluoroscopy, and 3D echocardiography (╪).

All procedures were performed under general anesthesia while using fluoroscopy and transesophageal echocardiography guidance. In the setting of COMBO, first the repair with either Cardioband (Figures 1A–E), CMCS (Figures 1F–H), or Neochord (Figures 1I, J) was done, followed by M-TEER in the same procedure. All procedures were carried out by the same first operator (SvB), with an overall experience for transcatheter mitral valve interventions of 380 procedures at the beginning, and 710 procedures at the end of the defined study timeframe. The details of each procedure have been reported previously (12, 15, 18–20).

### Echocardiographic examinations

Transthoracic echocardiography (TTE) was performed at baseline and at around 1 month [median 43 days (range 5–85)] and around 12 months [median 359 days (range 255–862)] after the procedure. Echocardiography images were taken by the cardiologists in the echocardiography lab and evaluated by two interventional echocardiography with high experience (HY and TFR). All subjects underwent standard 2-dimensional B-mode and Doppler TTE. All measurements were performed in accordance with the recommendations of the American Society of Echocardiography (21, 22). Echocardiographic parameters measured as recommended by current guidelines or position papers included the grading of MR (23), and tricuspid regurgitation (TR) (24), as well as left ventricular end-diastolic diameter (LVEDD), left ventricular end-systolic diameter (LVESD), left ventricular end-diastolic volume (LVEDV), left ventricular end-systolic (LVESV), left atrial volume (LAV), and index (LAVi), interventricular septum diameter (IVSD), posterior wall thickness (PWT), and left ventricular ejection fraction (LVEF). The volumes for LA and LV were measured using the “Simpson’s Method of disks” (22).

The ultrasound machines used were iE33, and Epiq7C (Philips, Andover, MA, USA), and GE Vivid E95 (GE Healthcare, Chicago, IL, USA). Images were evaluated offline by HY and TFR using IntelliSpace Cardiovascular and QLAB (Philips).

### Study endpoint

We evaluated all-cause mortality for all patients based on the entries in patients’ records and data reconciliation with the Rhineland-Palatinate bureau of vital statistics up to January 1st, 2022. In patients with complete TTE follow-up, we also evaluated the change of New York Heart Association functional class (NYHA) and the change of BNP to assess heart failure, while reverse remodeling of left cardiac chambers was investigated by observing the change in LVEDD, LVESD, LVEDV, LVESV, LV mass, and LAV index, respectively. Data at around 1 month (30d) and at around 1 year (1Y) after the procedure were compared to baseline values.

### Statistical analysis

All data were collected from the records in our hospital. All-cause mortality was assessed using the Kaplan-Meier statistics. Due to the small sample size, all variables independent of their distribution are either expressed as median (Q1, Q3), or as numbers (percentage), as appropriate. Paired testing was done using the Wilcoxon-Test, while the χ2 test or Fisher’s exact test were used to compare categorical variables. A *p*-value of <0.05 was considered statistically significant. All statistical analyses were performed using IBM SPSS statistics version 27 (IBM Corp., Chicago, IL, USA) and EZR version 1.55 (Saitama Medical Center, Moroyama, Saitama, Japan).

## Results

### Baseline patient characteristics, echocardiographic data

From March 2015 to April 2018 36 patients were treated using the COMBO approach with M-TEER and another device. Of them, one patient without baseline TTE was excluded, resulting in 35 patients as all population. Adequate echo data was available for analysis at both follow-up dates in 13 patients (median age 79 years old, males in 61.5%). All patients were symptomatic with NYHA mainly III or IV, and median logistic EuroScore was 17.0% (EuroScore II 4.7%). Almost all patients had atrial fibrillation, were prescribed a beta-blocker (ß-blocker) and angiotensin-converting enzyme inhibitor (ACEi) or angiotensin II receptor blocker (ARB) at maximal tolerated dosages (Table 1). Baseline echocardiography showed pathological values for LVEDD [61.5 mm (56.3, 67.0)], LVESD [50.0 mm (42.3, 59.3)], LVEDV [162.2 mL (102.1, 215.5)], LVESV [101.6 mL (60.8, 153.9)], LVEF [35.7% (25.3, 47.5)], LV mass [250.2 g (205.3, 294.7)], and LAVi [49.4 mL (42.1, 76.8)], respectively. The MR was severe in all cases, and the etiology was SMR in 10, and PMR in three patients, respectively (Table 2).

**TABLE 1:** Baseline characteristics.

	All patients *n* = 35	Patients with complete follow up *n* = 13	*p*-value*
Age, years	77.0 (73.0, 80.0)	79.0 (69.0, 84.0)	0.815
Male, *n* (%)	23 (65.7)	8 (61.5)	0.999
Height, cm	170.0 (167.5, 176.0)	170.0 (158.0, 184.0)	0.692
Weight, kg	76.0 (68.0, 88.5)	78.0 (45.0, 120.0)	0.926
BMI	27.0 (23.5, 29.4)	25.9 (18.0, 40.0)	0.999
BSA, m^2^	1.92 (1.75, 2.02)	1.87 (1.42, 2.41)	0.798
**NYHA functional class**			
Class I	0 (0)	0 (0)	0.37
Class II	5 (14.3)	0 (0)	
Class III	25 (71.4)	10 (76.9)	
Class IV	5 (14.3)	3 (23.1)	
Logistic EuroScore, %	17.0 (12.1, 20.0)	14.8 (10.1, 23.6)	0.261
EuroScore II, %	4.79 (3.53, 6.48)	3.78 (3.10, 5.28)	0.329
**Past medical history**			
Hypertension, *n* (%)	25 (73.5)	9 (69.2)	0.999
Dyslipidemia, *n* (%)	17 (48.6)	5 (38.5)	0.746
Diabetes mellitus, *n* (%)	8 (22.9)	3 (23.1)	0.999
CKD, *n* (%)	17 (48.6)	4 (30.8)	0.338
AFib, *n* (%)	30 (85.7)	12 (92.3)	0.999
Previous PCI, *n* (%)	14 (40.0)	6 (46.2)	0.75
Previous CABG, *n* (%)	5 (14.3)	1 (7.7)	0.999
Pacemaker or ICD, *n* (%)	13 (37.1)	3 (23.1)	0.497
**Medication**			
Beta blocker, *n* (%)	30 (85.7)	13 (100)	0.304
ACEi or ARB, *n* (%)	30 (85.7)	13 (100)	0.304
Diuretics, *n* (%)	32 (91.4)	13 (100)	0.553
**Laboratory data**			
BNP, pg/mL	359.0 (239.5, 819.3)	345.0 (100.0, 4326.0)	0.937
Creatinine, mg/dL	1.19 (0.96, 1.48)	0.99 (0.79, 1.56)	0.322
**Procedure**			
M-TEER + CMCS, *n* (%)	26 (74.3)	7 (53.8)	0.372
M-TEER + CB, *n* (%)	5 (14.3)	4 (30.8)	
M-TEER + NeoC, *n* (%)	4 (11.4)	2 (15.4)	

Values are median (Q1, Q3), or *n* (%).

*Comparison of the patients with complete follow-up vs. the remaining patients of the cohort.

ACEi, angiotensin-converting enzyme inhibitor; AFib, atrial fibrillation; ARB, angiotensin receptor blocker; BNP, brain natriuretic peptide; BMI, body mass index; BSA, body surface area; CABG, coronary artery bypass graft; CB, Cardioband; CKD, chronic kidney disease; CMCS, Carillon Mitral Contour System; ICD, implantable cardioverter defibrillator; M-TEER, mitral transcatheter edge-to-edge repair; NeoC, NeoChord; NYHA, New York Heart Association; PCI, percutaneous coronary intervention; TTE, transthoracic echocardiography.

**TABLE 2:** Baseline echocardiographic data.

	All patients *n* = 35	Patients with complete follow up *n* = 13	*p*-value*
LVESD, mm	50.0 (42.3, 59.3)	48.0 (34.0, 53.0)	0.258
LVEDD, mm	61.5 (56.3, 67.0)	60.0 (55.0, 66.8)	0.559
LVESV, mL	101.6 (60.8, 153.9)	69.8 [41.3, 117.5)	0.425
LVEDV, mL	162.2 (102.1, 215.5)	131.2 (82.4, 212.8)	0.412
LVEF, %	35.7 (25.3, 47.5)	46.8 (32.8, 54.8)	0.323
IVSD, mm	10.4 (8.2, 12.0)	8.7 (8.2, 10.9)	0.609
PWD, mm	8.9 (8.2, 9.7)	8.9 (7.9, 9.8)	0.924
LV mass, g	250.2 (205.3, 294.7)	215.7 (185.0, 273.9)	0.372
RWT	0.30 (0.27, 0.40)	0.31 (0.28, 0.44)	0.643
LAV index, mL/m^2^	49.4 (42.1, 76.8)	50.0 (47.2, 77.2)	0.372
SMR	27 (77.1)	10 (76.9)	0.999
PMR	8 (22.9)	3 (23.1)	
Severity of TR	–	–	0.999
1	19 (54.3)	7 (53.8)	
2	9 (25.7)	3 (23.1)	
3	6 (17.1)	3 (23.1)	
4	1 (2.9)	0 (0)	
5	0 (0)	0 (0)	

Values are *n* (%), or median (Q1, Q3).

*Comparison of the patients with complete follow-up vs. the remaining patients of the cohort.

LVEF, left ventricular ejection fraction; LVESD, left ventricular end-systolic diameter; LVEDD, left ventricular end-diastolic diameter; LVESV, left ventricular end-systolic volume; LVEDV, left ventricular end-diastolic volume; IVSD, interventricular septum diameter; PWD, posterior wall diameter; LV mass, left ventricular mass; LAV, left atrial volume; MR, mitral regurgitation; PMR, primary mitral regurgitation; RWT, relative wall thickness; SMR, secondary mitral regurgitation; TR, tricuspid regurgitation; TTE, transthoracic echocardiography.

### Transcatheter interventions and study endpoints

**FIGURE 2:**
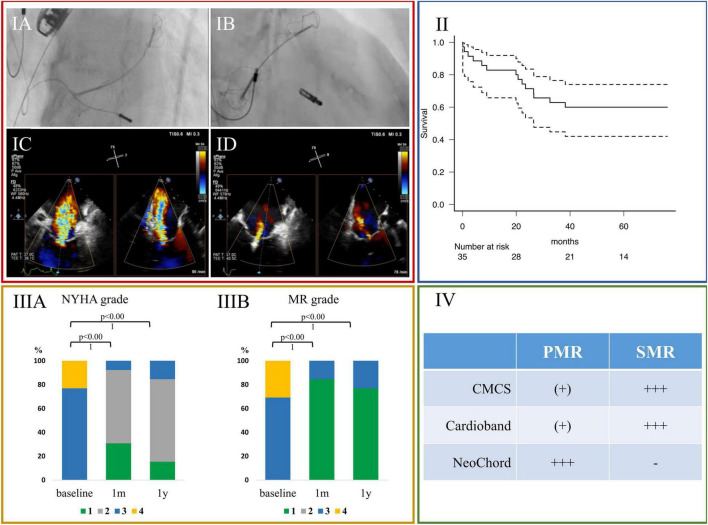
**(IA–ID)**: A representative case of severe SMR treated with M-TEER and CMCS. **(IA,IB)** Fluoroscopic images. **(IA)** At first CMCS was inserted into coronary sinus, followed by M-TEER **(IB)**. **(IC,ID)** Transesophageal echocardiographic images during procedure. **(IC)** Before CMCS was inserted. **(ID)** After both CMCS and M-TEER were implanted. The change of MR severity was from severe to mild. **(II)** Kaplan Meyer curve of all-cause death of all patients treated with COMBO-TMVr (*n* = 35). **(IIIA)** Serial New York Heart Association functional class, and **(IIIB)** serial severity of mitral regurgitation. **(IV)** Favorable ranges of use for different devices: (-); not applicable, [(+)]; useable, (+++) favorable use. CMCS, Carillon Mitral Contour System; LVEF, left ventricular ejection fraction; LVESV, left ventricular end-systolic volume; LVEDV, left ventricular end-diastolic volume; LV mass, left ventricular mass; LAVi, left atrial volume index; M-TEER, transcatheter edge-to-edge repair; MR, mitral regurgitation; NYHA, New York Heart Association.

The strategy used in these patients were M-TEER with either Cardioband (*n* = 4), CMCS (*n* = 7), or Neochord (*n* = 2), respectively. A representative case is shown in the Figures 2IA–ID.

In all 35 patients, survival rate was 94.3% (95% CI: 79.0–98.5) at thirty days, 82.9% (95% CI: 65.8–91.9) after 1 year, 71.4% (95% CI: 53.4–83.5) after 2 years, and 62.9% (95% CI: 44.8–76.5%) after 3 years, respectively (Figure 2II).

Compared to baseline, both NYHA functional class and MR severity were significantly improved at around 1 month and at around 1 year follow up in the patients with adequate follow-up (Figures 2IIIA, IIIB; *p* < 0.001, each).

**FIGURE 3:**
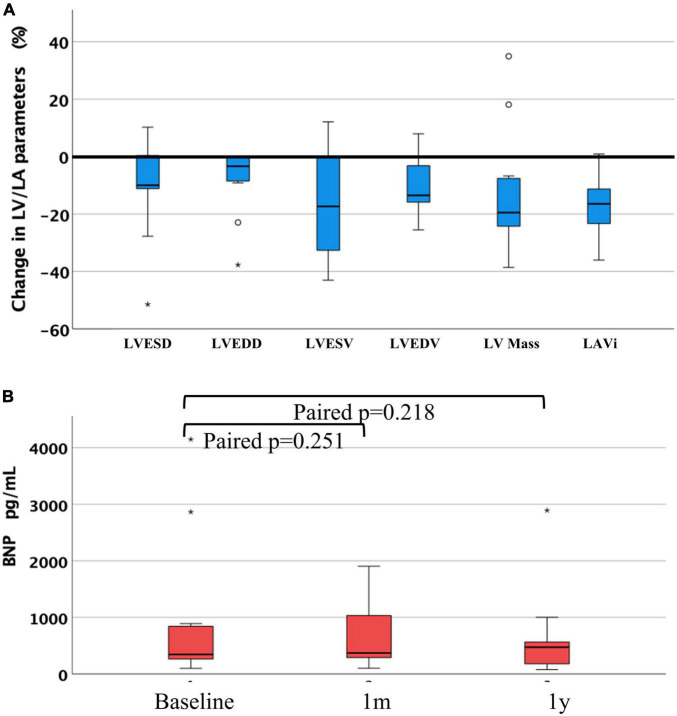
**(A)** Change ratio of left ventricular and left atrial parameters between at baseline and at 1 year after the procedure, and **(B)** difference of BNP at 1 month and at 1 year after the procedure compared with baseline. LVESD, left ventricular end-systolic diameter; LVEDD, left ventricular end-diastolic diameter; LVESV, left ventricular end-systolic volume; LVEDV, left ventricular end-diastolic volume; LV mass, left ventricular mass; LAVi, left atrial volume index; BNP, brain natriuretic peptide, “°” and “*” denote outliers.

We observed a reduction in LV dimensions, LV volumes and LV mass, respectively, as well as in LAVi, at both around 1 month and around 1 year. This significant reduction was sustained at both 1 month and 1 year follow-up when looking at LVESV, and LVEDV. Consequently, LVEF remained unchanged. Significant reduction in LV mass, and LAVi were observed only after 1 year compared to baseline, but not after 30 days (All Figure 3A, Table 3, and Supplementary Figure 1). When looking at the cases of SMR only, similar results were appreciated (Supplementary Figure 2).

**TABLE 3:** Serial echocardiographic data of left chamber.

	Baseline	1 month	Δ % (baseline–1 month)	Paired-*p*-value	1 year	Δ % (Baseline–1 year)	Paired-*p*-value
LVESD, mm	48.0 (18.4, 76.0)	38.0 (18.1, 73.0)	−4.0 (−14.2, 6.4)	0.382	43.0 (13.3, 68.0)	−9.9 (−11.1, 0.4)	0.045
LVEDD, mm	60.0 (44.0, 90.0)	55.0 (38.0, 79.0)	−9.1 (−16.4, −4.2)	0.008	58.0 (37.0, 81.8)	−3.3 (−8.5, 0.0)	0.009
LVESV, mL	69.8 (21.7, 392.0)	65.7 (12.4, 331.8)	−50.8 (−63.5, −33.4)	0.022	72.6 (16.6, 324.0)	−17.4 (−32.6, −0.4)	0.022
LVEDV, mL	131.2 (47.9, 485.5)	124.9 (43.3, 380.8)	−10.1 (−24.7, −2.8)	0.002	126.2 (51.7, 419.5)	−13.5 (−15.9, −3.2)	0.001
LV mass, g	215.7 (132.9, 507.3)	202.1 (98.1, 510.2)	−11.2 (−26.1, −5.8)	0.057	185.8 (100.7, 311.5)	−19.5 (−24.2, −7.6)	0.040
LAV index, mL/m^2^	50.0 (38.9, 134.8)	48.1 (23.1, 144.3)	−11.9 (−21.7, −5.4)	0.068	44.5 (24.9, 113.1)	−16.4 (−23.3, −11.3)	<0.001

Values are median (Q1, Q3). Abbreviations shown in Table 2.

Mitral valve mean inflow pressure was 2.3 mmHg (1.5; 3.0) at 30 days, and 3.0 mmHg (1.8; 4.6) at 1 year. Regarding BNP, there was a nominal reduction but no significant change at either follow-up compared to baseline (Figure 3B).

## Discussion

The findings of the present study indicate that the COMBO-TMVr strategy, using concomitant M-TEER with another approach to treat symptomatic MR seems feasible and could facilitate sustained reverse remodeling of the overloaded left cardiac chambers at 1 month and 1 year of follow up, offering a toolbox tailored to the patient’s needs.

First, reverse cardiac remodeling can be achieved by the use of guideline directed medical therapy (25, 26). In our study almost all patients were already prescribed ß-blockers and ACEi or ARBs at baseline at maximal tolerated dosages, including patients with PMR (23%). Second, TMVr used as MONO therapy has shown effectiveness in studies: data has been published on the reverse remodeling of the left atrium (18), and of the left ventricle in spite of severe LV dilatation (13) when using the CMCS for symptomatic MR. Regarding Cardioband, a prior study showed that mean LVEF was decreased 37–31%, and LV volumes were not significantly altered at 6 months after the procedure (mean LVEDV index: 93.4–89.2 mL) (27). Moreover, Gonçalves-Teixeira et al. (28) also showed reverse remodeling of the LV and the LA after using TMVr with NeoChord in patients with symptomatic severe PMR. With the limitation that their study follow-up was 6 months, the results seem compareable to ours (28). There are many studies on M-TEER and reverse remodeling to date. A significantly positive LV change using M-TEER was described for all entities of MR combined, as well as SMR and PMR, respectively, at 6 months and 1 year follow-up (29–33). However, there is also data from the COAPT trial showing that 1 year after M-TEER in SMR patients with a disproportionate phase of MR, LVESV, LVEDV and LAV increased (6.5 ± 3.9, −5.1 ± 4.5, and 9.7 ± 2.4 mL, respectively), while LVEF decreased over time (−5.6 ± 1.2%) (34). Although the study indicated that M-TEER was implanted in 95% of the included patients and could keep the reduction of MR, leading to the significantly better clinical outcomes compared with the medical therapy (5), a sustained reverse remodeling of the left ventricle and the left atrium could not be achieved (34). Hence, optimal patient and therapy selection seem to play a pivotal role.

Our study, which included patients with M-TEER plus other devices, indicated a sustained significant reverse remodeling of the left cardiac chambers. However, it is unclear why the combination with two devices with different mechanisms could produce this effect. One might speculate that the COMBO approach may be a better option for patients suffering from severe MR with a selected anatomy, where the devices combined could best play out their advantages (Figure 2IV). For example, SMR due to the combined pronounced dilatation of the mitral annulus and leaflet tethering might be addressed more completely when using strategies targeting the dilated annulus—e.g., by using CMCS or Cardioband—in combination with M-TEER, targeting the insufficient leaflet coaptation. In these scenarios, it makes sense to perform annuloplasty first, as it facilitates leaflet approximation by its own, thus empowering the following M-TEER to achieve a more potent leaflet coaptation. In PMR, due to excessive flailing leaflet tissue, the use of Neochord can first correct the damaged leaflet suspension, while a successive M-TEER would then address the remaining MR. There is a discrepancy in our study when looking at the time reverse remodeling takes for LA, and LV, each. The reductions in LVEDV are significant as soon as 30 days, but for the LAVi this is only apparent at 1 year. There is an absolute reduction of the LAVi at 30 days of almost 12 mm. The lacking significance here might be caused by the low number of patients in the study, as suggested by the wide IQR (LAVi-reduction of 11.9 mm (−21.7, −5.4). Furthermore, most of the patients were suffering from atrial fibrillation, distinctively causing LA remodeling.

Combining more than one (transcatheter) therapy in one procedure could likely increase risks, especially in an already high-risk population. We found that both 30 days mortality, and long term mortality of the patients in our study seemed comparable to the data of the individual procedures. At the same time, the patient population treated seemed either also at comparable, or even at higher risk then those treated with the individual TMVr alone (5, 12, 35–38).

To the best of our knowledge, there are up to now no original studies or review articles concerned with the COMBO-TMVr, only case reports. Of these, all but one are focusing on “staged,” but not on concomitant therapy (8–11). Therefore, this study offers the perspective that the use of a COMBO strategy could act as a toolbox facilitating an individual and integrated therapy approach as opposed to “one size fits all.”

## Limitations

This study has several limitations. First, this is a retrospective and single center study. Hidden confounders could be present, and a selection bias cannot be ruled out, especially with one of the key messages of this work being “proper patient selection.” Second, these errors might be aggravated by the low number of patients treated with the COMBO approach. However, the COMBO approach itself is a very rare procedure. For echocardiographic analysis, the number was further reduced by 22 patients. Several reasons accounted for this 63% loss-to-follow-up: six patients did not survive, nine patients came from outside the geographical area, and seven patients canceled their appointment. The third main limitation is that the echocardiographic data was evaluated not by a core laboratory center, but in our institution, making external validity possibly difficult. Still, the echo data were evaluated by only two cardiologists with high experience from high-volume centers for TMVr. Fourth, there is no control group in this study. Therefore, it remains unclear whether COMBO-TMVr itself caused the reverse remodeling, and if so, how much the additive effect is to MONO therapy. Fifth, the COMBO-TMVr group is not homogenous, with three different “partners” to M-TEER, i.e., CMCS, Cardioband, and NeoChord, used in both PMR and SMR. Although we tried to investigate the pure effect COMBO-TMVr as close as possible by excluding patients that were treated with a “staged” therapy, a focused prospective study design looking specifically at one entity of MR treated with one COMBO-TMVr variant would be desirable. Hence, this study can only serve and should be interpreted as “hypothesis generating.” Finally, the study does not follow the clinical course of the patients more than 1 year after the procedure, and the effect of the combination therapy on clinical outcomes directly is unknown.

## Conclusion

A COMBO approach seems feasible in selected high-risk patients suffering from symptomatic MR, possibly offering a toolbox for a therapy tailored to the patient’s needs in order to achieve reverse remodeling with significant volume reduction of left cardiac chambers during 1 year after the procedure. However, with the COMBO approach remaining a rare therapy concept at this time, the positive results of this retrospective analysis need further validation.

## Data availability statement

The original contributions presented in this study are included in this article/supplementary material, requests to access the datasets should be directed to corresponding author.

## Ethics statement

The studies involving human participants were reviewed and approved by “Ethik-Kommission der Landesärztekammer Rheinland-Pfalz”, approval 2019-14692. Written informed consent for participation was not required for this study in accordance with the national legislation and the institutional requirements.

## Author contributions

HY: writing manuscript and data acquisition. TFR: writing manuscript, coordinating and overseeing project, conducting procedures, and echo guidance. MG and AT: review manuscript. JD and TG: review manuscript and echo guidance. JZ and BS: data acquisition. TM: overseeing project. RSvB: overseeing project and conducting procedures. All authors contributed to the article and approved the submitted version.
