# Supplementary figures and images for: Reverse cardiac remodeling in patients undergoing combination therapy of transcatheter mitral valve repair
Source: Front Cardiovasc Med. 2023 Feb 15;10:1029103. doi: 10.3389/fcvm.2023.1029103 (PMC9975952; doi:10.3389/fcvm.2023.1029103)

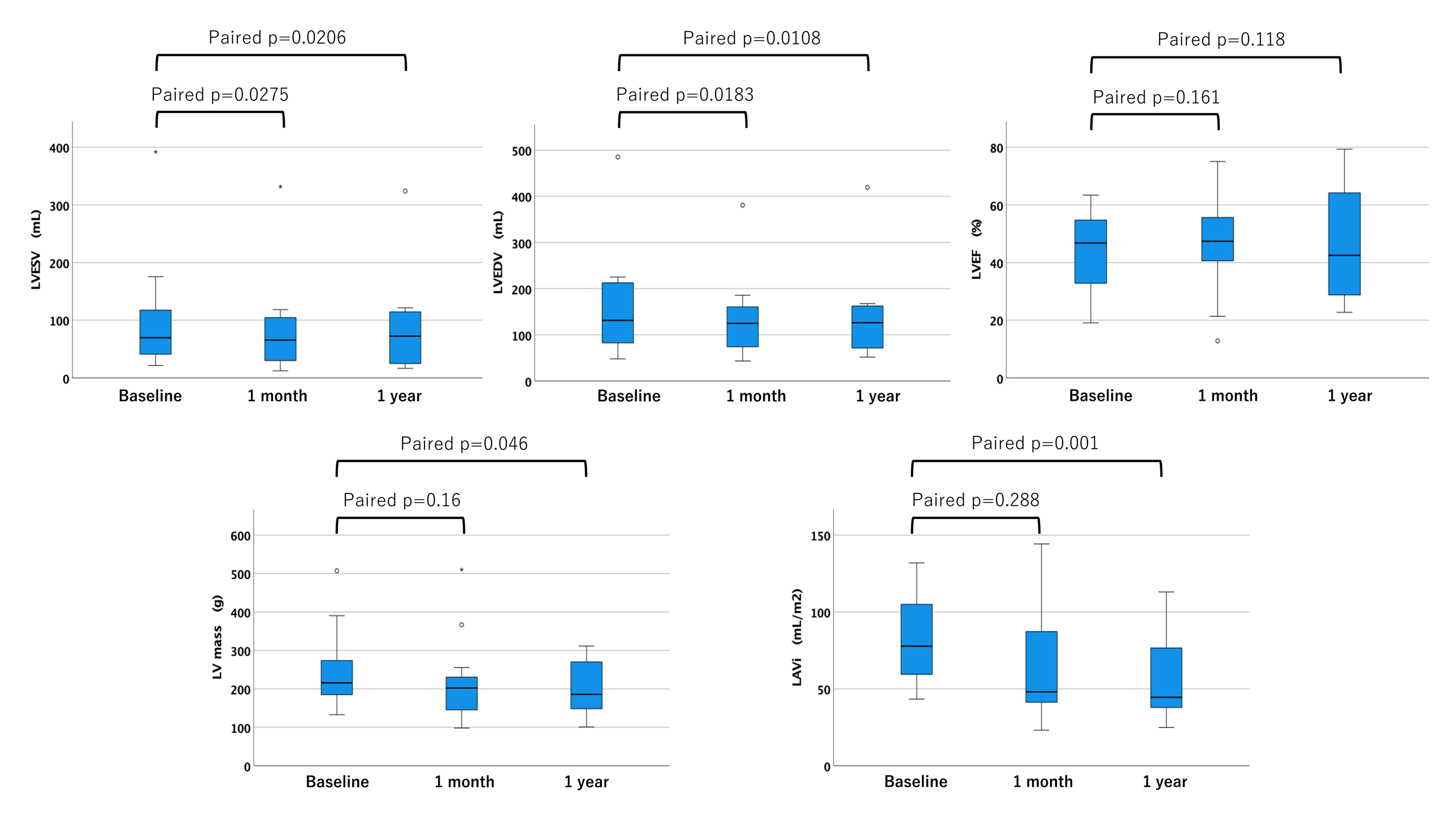

Supplement: Supplementary file 1 [file Image_1.jpeg]

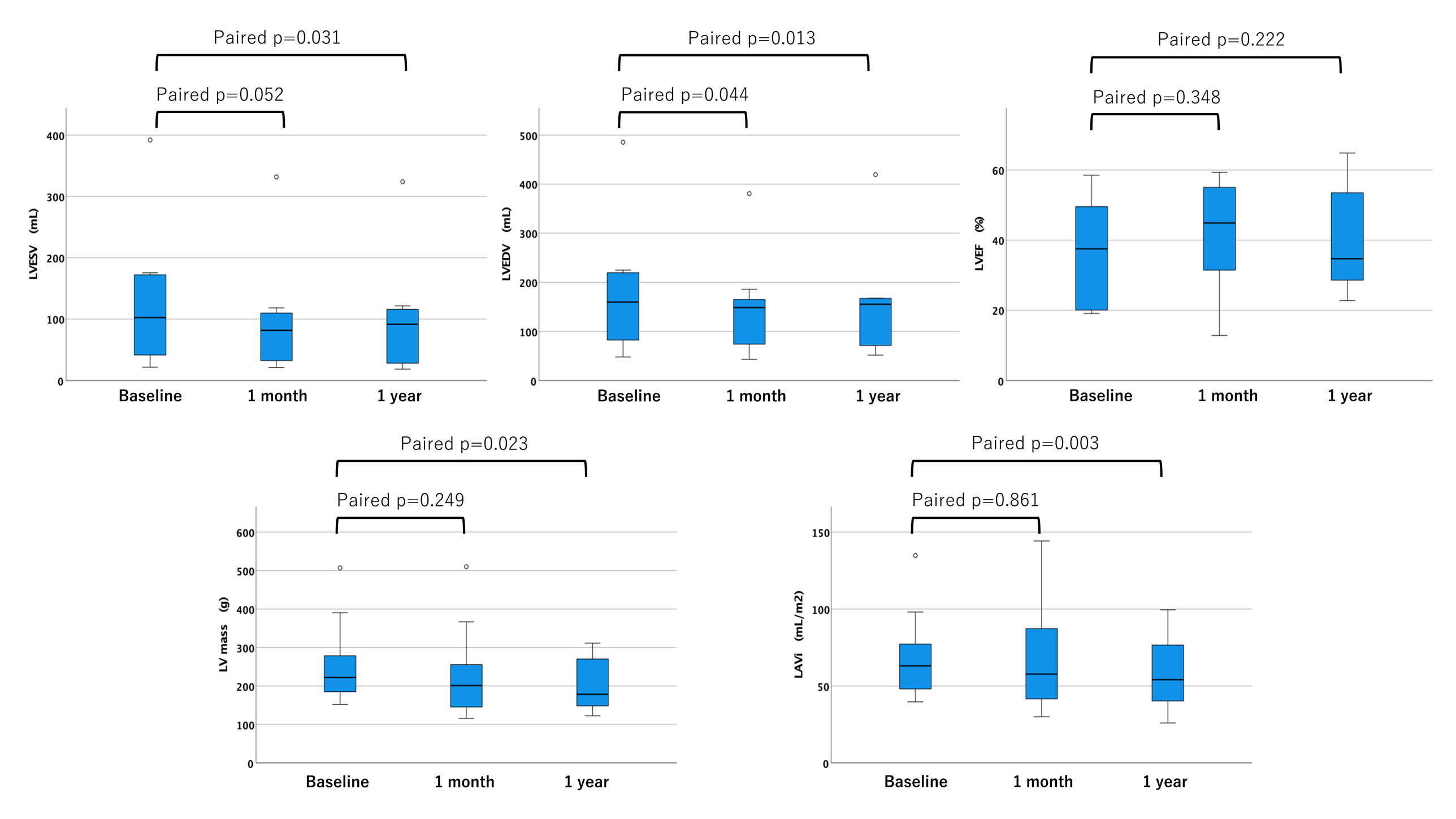

Supplement: Supplementary file 2 [file Image_2.jpeg]
